# Use of traditional medicines in the management of HIV/AIDS opportunistic infections in Tanzania: a case in the Bukoba rural district

**DOI:** 10.1186/1746-4269-3-29

**Published:** 2007-07-10

**Authors:** Daniel P Kisangau, Herbert VM Lyaruu, Ken M Hosea, Cosam C Joseph

**Affiliations:** 1 Department of Botany, University of Dar es Salaam, PO Box 35060, Dar es Salaam, Tanzania; 2 Department of Chemistry, University of Bielefeld, PO Box 10 01 31 – 3350, Bielefeld, Germany; 3 Department of Molecular Biology and Biotechnology, PO Box 35060, University of Dar es Salaam, Dar es Salaam, Tanzania; 4 Department of Chemistry, University of Dar es Salaam, PO Box 35061, Dar es Salaam, Tanzania

## Abstract

**Background:**

Ethnobotanical surveys were carried out to document herbal remedies used in the management of HIV/AIDS opportunistic infections in Bukoba Rural district, Tanzania. The district is currently an epicenter of HIV/AIDS and although over 90% of the population in the district relies on traditional medicines to manage the disease, this knowledge is impressionistic and not well documented. The HIV/AIDS opportunistic conditions considered during the study were Tuberculosis (TB), Herpes zoster (Shingles), Herpes simplex (Genital herpes), Oral candidiasis and Cryptococcal meningitis. Other symptomatic but undefined conditions considered were skin rashes and chronic diarrhea.

**Methods:**

An open-ended semi-structured questionnaire was used in collecting field information. Descriptive statistics were used to analyze the ethnobotanical data collected. Factor of informant consensus (F_ic_) was used to analyze the ethnobotanical importance of the plants.

**Results:**

In the present study, 75 plant species belonging to 66 genera and 41 families were found to be used to treat one or more HIV/AIDS related infections in the district. The study revealed that TB and oral candidiasis were the most common manifestations of HIV/AIDS opportunistic infections affecting most of the population in the area. It unveils the first detailed account of ethnomedical documentation of plants focusing the management of HIV/AIDS related infections in the district.

**Conclusion:**

It is concluded that the ethnopharmacological information reported forms a basis for further research to identify and isolate bioactive constituents that can be developed to drugs for the management of the HIV/AIDS opportunistic infections.

## Background

According to WHO [1], traditional medicine continues to provide health coverage for over 80% of the world population, especially in the developing world. In many African countries including Tanzania, traditional healers play a crucial role of providing primary health care including taking care of people living with emerging diseases such as HIV/AIDS [2,3]. In 2006, almost two thirds (63%) of all persons infected with HIV/AIDS in the world are living in sub-Saharan Africa [4]. HIV/AIDS pandemic is currently the most socio-economic challenge that faces Tanzania as it affects mostly the young and most economically productive population [5]. This translates to loss of skills, talents, expertise and man-hours. Majority of the people living with HIV/AIDS are susceptible to fungal and bacterial opportunistic infections that result from immunosuppression [Bii, 2001-unpublished abstract]. These infections have been reported from the early days of the HIV/AIDS pandemic [6] and are one of the leading causes of deaths in Tanzania and worldwide [7]. Treatment of such infections is therefore one of the most important factors for management of HIV/AIDS cases. However, poverty, high cost of life-enhancing drugs, resistance to conventional medicine and the serious side effects associated with antiretroviral drugs are the main draw backs to the use of conventional therapies.

More than 60% of the population in Tanzania depends on traditional medicines for the management of various diseases including HIV/AIDS [8]. Due to scarcity of drugs, many people living with HIV/AIDS opt for traditional health services for the control of the disease. Besides, the Lake Victoria basin which harbors the study area and the Great lakes region of East and Central Africa are now considered part of the global epicenter for HIV/AIDS, with 50% of bed occupancy in hospitals with AIDS patients in the mid-term to terminal stages of the disease [Aduma, 2001-unpublished abstract]. Furthermore, in this region more than any other in Tanzania, the HIV/AIDS pandemic has had the worst impact as it was the first to show a significant number of cases in the early 1980's, so that the disease has had the longest history in the region [5,9]. It is therefore reasonable to assume that the devastating impact of HIV/AIDS pandemic in the region and in Bukoba rural district in particular, coupled with the severe shortage of health personnel might have forced the inhabitants to develop coping mechanisms by adopting alternative sources of primary health care, one of which has been the use of herbal therapies.

Even though there are a good number of reports on traditional uses of plants to treat various diseases in the country, knowledge on herbal remedies used to manage HIV/AIDS in particular is scanty, impressionistic and not well documented. Consequently, this paper presents the first detailed account of the status and use of traditional medicines in the management of HIV/AIDS opportunistic infections in Tanzania.

## Methods

### The study area

**Figure 1:**
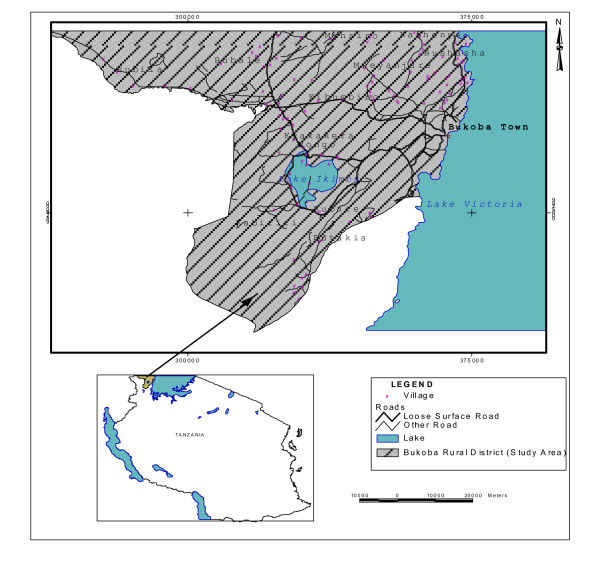
A map showing the location of Bukoba rural district in Tanzania.

Bukoba rural district (Fig. 1) is among six administrative districts forming Kagera region in the Lake Victoria Basin in Tanzania. The district borders Uganda to the North, Lake Victoria to the East, Waters of Mara region to the South, Muleba district to the South East and Karagwe district to the West. It is composed of 168 villages in 41 wards and 6 divisions, and a total population of 395,130 [9]. The district is predominantly occupied by the Haya tribe who speak *Kihaya *language. Agriculture is the economic mainstay of the district and accounts for 50% of the Region's Gross Domestic Product (GDP). The main crops are green bananas, coffee, beans, cotton and cassava. Other crops include sugarcane, sweet potatoes, vegetables, millet and sorghum [9]. The population-Doctor ratio stands at 95,000:1, the lowest of all the six districts in Kagera region [5,9], indicating a severe inadequacy of health personnel.

### Ethnobotanical surveys

Ethnobotanical surveys were carried out in four out of the six divisions in the district. These were Kiamtwara, Misenye, Katerero and Rubale divisions. With a Prior Informed Consent (PIC), authentic and well known herbal practitioners were identified through Participatory Rural Appraisal (PRA) approach, with the assistance of local administrative officers. They were then interviewed using semi-structured open-ended questionnaires [10]. Interviews were conducted in the local *Kihaya *language except for a few cases where the respondents were erudite and could understand Kiswahili. Information regarding the local names of the plant species, parts used, preparation, administration and the disease condition treated was documented as shown in table 1. The practitioners were also used as guides in field excursions to collect plant voucher specimens which were identified by Mr. Suleiman Haji and Mr. Frank Mbago of the University of Dar es Salaam, Department of Botany. The voucher specimens were later coded and deposited at Department of Botany herbarium, University of Dar es Salaam (Table 1). Factor of informant consensus (F_ic_) was used to analyze the ethnobotanical importance of the reported plant species according to Schlage *et al*. [11] and Owuor and Kisangau [12]. F_ic _gives the relationship between the number of use-reports in each category (n_ur_) and number of taxa used (n_t_):

F_ic _= (n_ur _- n_t_/n_ur _- 1)

**Table 1:** Plant species used in treating various HIV/AIDS related conditions in Bukoba rural district

**Family**	Plant name	**Local name (Haya)**	**Part used**	Condition treated	**Collection code No.**
Acanthaceae	*Thurnbergia alata *Sims	Rwankura	Leaves/Roots	Oral candidiasis	DK013/06
Aloaceae	*Aloe sp*.	Enkaka	Leaves	Herpes zoster	DK046/06
Anacardiaceae	*Mangifera indica *L.	Omunembe	Leaves	Tuberculosis (TB)	DK037/06
	*Ozoroa insignis *Del.	Omukerenge	Roots	Skin rashes, Tuberculosis, Herpes simplex, Herpes zoster, Cryptococcal menengitis, Oral candidiasis	DK023/06
	*Rhus natalensis *Krauss	Omusheshe	Leaves/Roots	Herpes zoster, Herpes simplex, Cryptococcal meningitis, skin infections	DK044/06
	*Rhus vulgaris *Meikle	Omukanja	Leaves/Roots	Chronic diarrhea, skin rashes	DK036/06
	*Pseudospondias microcarpa *Engl.	Omuziru	Leaves/Bark	Tuberculosis, Oral candidiasis	DK005/06
	*Lannea schimperi *(A. Rich) Engl.	Ombumbo	Bark	Tuberculosis, Skin rashes, Herpes zoster, Herpes simplex, Chronic diarrhea	DK047/06
Annonaceae	*Annona senegalensis *Pers.	Omukonya	Root	Herpes zoster, Cryptococcal meningitis, Skin infections	DK034/06
Apocynaceae	*Rauvolfia vomitoria *Afz.	Omunyabusindi	Leaves/Bark/Roots	Herpes zoster, Herpes simplex, Skin rashes.	DK030/06
Araliaceae	*Cussonia arborea *Hochst. Ex A. rich	Kijagaajaga	Bark	Chronic diarrhoea	DK022/06
Asteraceae	*Vernonia adoensis *Walp.	Nyakibasi	Leaves	Tuberculosis	DK008/06
	*Vernonia amygdalina *Del.	Omumbilizi	Leaves	Skin rashes, Chronic diarhhoea, Herpes zoster, Herpes simplex, Cryptococcal meningitis.	DK016/06
	*Senecio syringifolius *O. Hoffm.	Ekishenda	Roots	Herpes simplex	DK031/06
	*Ageratum conyzoides *L.	Kyabakiriao	Leaves	Cryptococcal meningitis, Herpes zoster.	DK025/06
	*Bidens pilosa *L.	Mbukurura	Leaves	Oral candidiasis.	DK054/06
	*Conyza floribunda *H.B.K.	Lukobe	Leaves	Skin rashes	DK027/06
Bignonaceae	*Kigelia africana *(Lam.) Benth.	Omujunguti	Bark/Fruit	Herpes simplex	DK032/06
Caesalpiniaeae	*Cassia abbreviate *Oliv.	-	Leaves	Skin rashes	DK045/06
	*Senna occidentalis *(L.) Link	Mwita njoka	Roots	Chronic diarrhea	DK021/06
	*Cassia mimosoides *L.	Akashanganziru	Leaves/Roots	Tuberculosis	DK024/06
Capparaceae	*Capparis erythrocarpos *Isert	Oluvuranganga	Roots	Skin rashes, Tuberculosis, Cryptococcal meningitis, Oral candidiasis, Herpes zoster, Herpes simplex, chronic diarrhoea	DK028/06
	*Gynadropsis gynandra *(L.) Briq.	Eiopyo	Leaves	Oral candidiasis, Oral sores	DK033/06
	*Capparis tomentosa *Lam.	Omukolokomba/Rukwatango	Roots	Tuberculosis, Oral candidiasis, Herpes zoster, Herpes simplex	DK020/06
Caricaceae	*Carica papaya *L. (male)	-	Leaves/Roots	Oral candidiasis	DK035/06
Celastraceae	*Maytenus senegalensis *(Lam.) Exell	Omunyambuliko	Bark/Root	Herpes simplex, Herpes zoster, Oral candidiasis, Skin rashes, Tuberculosis	DK018/06
Chenopodiaceae	*Chenopodium opulifolium *Koch. & Ziz.	Mwitango	Leaves	Herpes simplex	DK015/06
	*Chenopodium Ambrosioides *L.	Akaita malogo	Leaves	Herpes simplex, cryptococcal meningitis	DK056/06
Chrysobalanaceae	*Parinari curatellifolia *Benth.	Omunazi	Bark/Root	Skin rashes, Tuberculosis, Chronic diarrhea, Herpes zoster, Herpes simplex.	DK039/06
Clusiaceae	*Garcinia buchananii *Bak.	Omusharazi	Bark/Root	Tuberculosis, Chronic diarrhoea, Cryptococcal Meningitis, Herpes zoster, Herpes simplex, Skin rashes	DK063/06
	*Psorospermum febrifugum *Spach.	Ekiana	Bark/Root	Herpes zoster, Herpes simplex, Cryptococcal meningitis, Skin infections.	DK003/06
	*Harungana madagascariensis *Lam. Ex Poir	Omujumbo	Leaves/Bark	Chronic diarrhea	DK006/06
Combretaceae	*Combretum collinum *Sound.	Omukoyoyo	Leaves/Bark/Roots	Chronic diarrhea, Tuberculosis	DK041/06
	*Terminalia mollis *Laws	Muhongora	Bark	Cryptococcal meningitis, Tuberculosis	DK058/06
Convolvulaceae	*Ipomoea sinensis *(Desr.) Choisy	Omusinda nyungu	Leaves	Oral candidiasis, Tuberculosis	DK055/06
Cucurbitaceae	*Zehneria scabra *(L.f.) Sond.	Akabindizi	Whole plant	Cryptococcal meningitis, Oral candidiasis, Skin rashes, Herpes simplex.	DK017/06
Dennstaedtiaceae	*Pteridium aquilinum *(L.) Kuhn.	Olulele	Leaves	Oral candidiasis, Tuberculosis	DK029/06
Dracaenaceae	*Dracaena steudneri *Engl.	Omugorogoro	Bark	Cryptococcal meningitis, Tuberculosis, Oral candidiasis	DK014/06
Euphorbiaceae	*Sapium ellipticum *(Krauss) Pax	Omushasha	Bark	Tuberculosis, Herpes zoster, Cryptococcal meningitis	DK019/06
	*Ricinus communis *L.	Omujuna	Roots	Chronic cough	DK048/06
	*Jatropha curcas *L.	Ekiyo	Leaves	Skin rashes, Oral candidiasis	DK011/06
	*Antidesma venosum *Tul.	Mbatabata	Roots	Tuberculosis, Chronic diarrhoea, Oral candidiasis	DK049/06
	*Phyllanthus reticulatus *poir.	Kaumura	Leaves	Herpes simplex	DK076/06
Lamiaceae	*Plectranthus barbatus *Andr.	Kasindano/Kishwija	Leaves	Oral candidiasis, Herpes zoster, Herpes simplex, Skin rashes	DK010/06
	*Plectranthus comosus *Sims	Mukono wa nkanda	Leaves	Herpes zoster, Herpes simplex, Skin rashes, Oral candidiasis, Tuberculosis	DK071/06
	*Ocimum gratissimum *L.	Kashwagara	Leaves	Chronic diarrhea, Herpes simplex	DK065/06
Malvaceae	*Hibiscus fuscus *Garcke	Olushuya	Leaves	Chronic diarrhoea	DK053/06
Mimosaceae	*Entada abyssinica *A. rich.	Mwiganjura	Leaves/Bark	Skin rashes, Tubercuilosis, Oral candidiasis, Herpes zoster, Herpes simplex.	DK026/06
	*Entada leptostachya *Steud ex A. rich.	Ekitakuli	Roots	Skin rashes, Tuberculosis, Herpes simplex, Herpes zoster	DK043/06
	*Acacia hockii *De Willd.	Mugando	Bark	Herpes zoster	DK038/06
Myricaceae	*Myrica salicifolia *A. Rich.	Omukikimbo	Bark/Root	Tuberculosis, Chronic diarrhea, Cryptococcal meningitis, Herpes simplex	DK057/06
Myrtaceae	*Psidium guajava *L.	Omupera	Leaves	Tuberculosis, Chronic diarrhea	DK042/06
	*Syzygium guineense *(Willd) DC	Omuchwezi	Bark	Chronic diarrhea	DK059/06
	*Syzygium cordatum *Krauss	Omugege	Leaves/Bark	Herpes zoster, Herpes simplex, Skin rashes	DK070/06
Olacaceae	*Ximenia americana var. caffra *(Sond.) Engl.	Omusheka	Roots	Skin rashes	DK074/06
Papaveraceae	*Argemone mexicana *L.	Akatojo	Leaves/Seeds	Cryptococcal meningitis	DK062/06
Papillionaceae	*Erythrina abyssinica *DC.	Omurinzi	Bark/Root	Tubeculosis	DK040/06
	*Eriosema psoraleoides *(Lam.) G. Don.	Omukakara	Leaves	Chronic diarrhea	DK077/06
	*Abrus precatorius *L.	Kaligaligo	Leaves	Oral candidiasis	DK052/06
	*Cajanus cajan *(L.) Millsp.	Mtandaikwa	Stem string	Oral candidiasis	DK066/06
Passifloraceae	*Adenia gummifera *(Harv) Harms.	Nyarimari	Stem/Root	Oral candidiasis	DK073/06
Phytolacaceae	*Phytolacca dodecandra *L'Herit	Muhoko	Leaves	Herpes zoster, Skin rashes.	DK079/06
Polygalaceae	*Securidaca longipedunculata *Fres.	Omweiya	Leaves/Bark/Root	Cryptococcal meningitis, Oral candidiasis	DK069/06
Polygonaceae	*Rumex usambarensis *(Dammer) Dammer	Akarurinzi	Leaves/Roots	Chronic diarrhea, Oral candidiasis, Skin infections	DK060/06
Ranunculaceae	*Clematis hirsute *Guill. & Perr.	Omnkamba	Leaves	Tuberculosis, Cryptococcal meningitis, Herpes zoster	DK051/06
Rubiaceae	*Canthium zanzibarica *Klotzsch.	Omushangati	Bark/Root	Cryptococcal meningitis	DK080/06
	*Tarenna graveolens *(S.Moore) Breun.	Omushangati	Bark/Roots	Cryptococcal meningitis	DK067/06
	*Vagueria infausta *Hochst.	Mubungo	Leaves	Oral candidiasis	DK061/06
Rutaceae	*Citrus limon *(L.) Burm.f.	-	Root	Tuberculosis	DK075/06
Sapindaceae	*Allophyllus africanus *Beauv.	Katatera Mnyanya	Leaves	Chronic diarrhea	DK050/06
Tiliaceae	*Grewia bicolor *Juss	Omukoma	Leaves/Bark/Roots	Chronic diarrhea	DK064/06
Ulmaceae	*Trema orientalis *(L.) Blume	Muuwe	Leaves	Oral candidiasis	DK078/06
Verbenaceae	*Vitex fischeri *Gurke	Omuunda	Bark	Herpes zoster, Tuberculosis, Herpes simplex, Skin rashes	DK068/06
Vitaceae	*Rhoicissus tridentate *(L.f.) Wild & Drum.	Ekimara	Leaves	Herpes zoster	DK072/06

### Knowledge on HIV/AIDS opportunistic infections

During the interviews, the symptoms of various HIV/AIDS opportunistic infections were described to the healers so as to enable them give the appropriate plant species they usually use to manage the infections [13,4-16] (Table 2). The Opportunistic infections considered in the present study were Tuberculosis (TB) locally called *Ndwala enkuri*, Oral candidiasis (*Mbunya kanua*), Cryptococcal meningitis (*Mulalamo*), Herpes zoster [Shingles] and Herpes simplex [Genital herpes] (*Ebiere*). The symptomatic conditions, skin rashes and chronic diarrhea are locally called *Ubwere *and *Kuaruka *respectively.

**Table 2:** Symptoms of HIV/AIDS opportunistic infections described to the traditional healers during the interviews

**Disease condition**	**Symptoms**
Tuberculosis	Persistent or chronic cough, Mucopurulent sputum, recurring dull, aching pain or tightness in the chest and Dyspnea
Oral candidiasis	Oral thrush, Oral mucosal lesions, mouth ulcers and difficulty in swallowing
Cryptoccocal meningitis	Fever, frequent headache, mental confusion, seizures, malaise and fatigue
Herpes zoster	Localized burning sensation, reddening of the skin followed by the appearance of grouped, dense blisters (Vesicles) and sores on the skin
Herpes simplex	Mouth sores, genital lesions, ulcers or blisters

## Results

**Figure 2:**
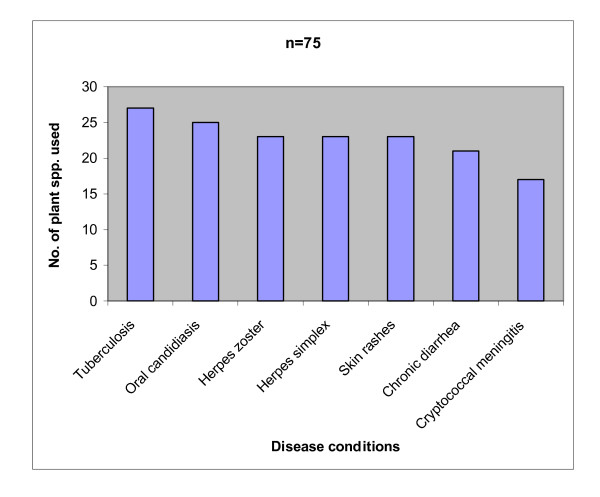
Disease conditions versus the number of plant species used to treat them.

A total of thirty herbal practitioners aged between 32 and 80 years of age were interviewed during the study. Twenty two out of the thirty respondents (73%) were above 50 years of age. Twenty one of these were women and only nine were men, constituting a percentage of 70% and 30% respectively. Majority of the respondents were peasant farmers and non- educated. It was found that most informants could unambiguously characterize symptoms of the targeted HIV/AIDS opportunistic infections without much problem. During the study, 75 plant species in 66 genera and 41 families were known to be used to treat one or more of the reported HIV/AIDS related infections in the district. The families Anacardiaceae, Asteraceae, Capparaceae, Clusiaceae, Euphorbiaceae, Papillionaceae, Rubiaceae, Myrtaceae, Mimosaceae and Lamiaceae constituted 52% of all the reported plant species, with each family having three or more species associated with the treatment of the opportunistic infections documented. The highest number of plant species used to treat the various conditions was recorded for TB which had 27 of the 75 documented species. It was followed by oral candidiasis with 25, Herpes zoster (23), H. simplex (23), skin rashes (23) chronic diarrhea (21) and cryptococcal meningitis (17) (Fig. 2). Thirty five of the 75 plant species were used to manage only one of the seven conditions reported, 39 were used to manage two up to six of the conditions, while one plant species only, *Capparis erythrocarpos *was used to treat all the seven reported disease conditions.

There were a total of 249 independent informant reports on plant usage against the various reported conditions. The total number and the percentage informant reports for each condition are shown in table 3.

**Table 3:** Percentage informant reports on plant usage against the various conditions

**Condition**	**Number of reports**	**Percentage**
Herpes zoster	50	20%
Skin rashes	42	17%
Tuberculosis	38	15%
Herpes simplex	34	14%
Oral candidiasis	33	13%
Cryptococcal meningitis	30	12%
Chronic diarrhoea	22	9%
Total	249	100%

**Figure 3:**
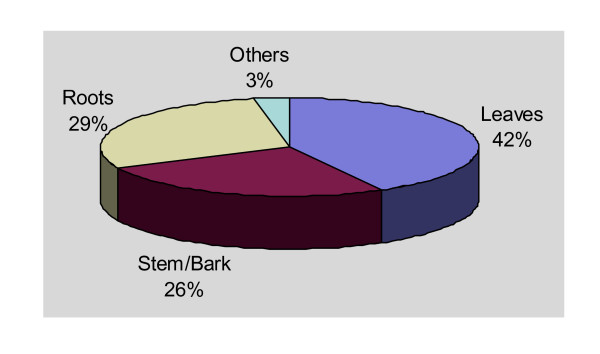
Percentage use of plant parts.

The study revealed that leaves were the most popular parts used in preparing herbal remedies and comprised 42% of all the reports on use of plant parts. This was followed by roots (29%), stem or bark (26%) and other parts of plants like fruits or seeds (3%) (Fig. 3). Most of these plant parts were harvested unsustainably without putting any consideration for future resource availability. For example, there was evidence of total ring barking of trees, total uprooting or cutting of the whole plant.

**Figure 4:**
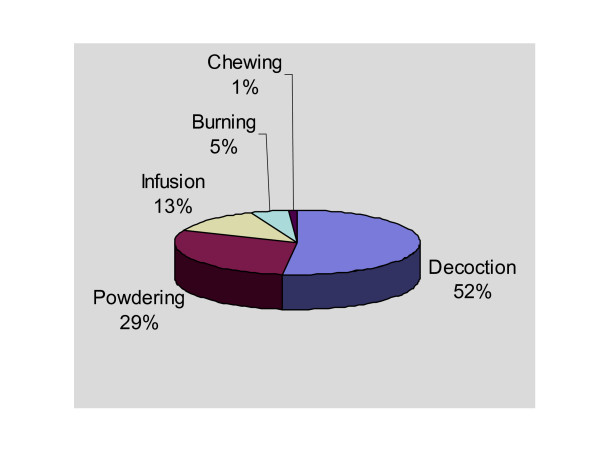
Percentage forms of herbal preparations.

Different methods were employed in preparing and dispensing herbal remedies as shown in table 4 and Fig. 4.

**Table 4:** Percentage forms of preparing herbal remedies

**Method of preparation**	**Percentage**
Boiling (Decoctions)	52%
Drying in sun and pulverization	29%
Soaking in cold water (Infusion)	13%
Burning	5%
Chewing	1%

The most common method of drug administration was by oral application of decoctions or infusions, especially for conditions like TB, Herpes zoster, H. simplex and Cryptococcal menengitis. Powdered medicines were mixed with jelly and applied as ointments for skin eruptions. Alternatively, decoctions or infusions were bathed with for the skin infections. Licking was especially employed for oral infections like oral candidiasis.

The use of the Factor of informant consensus (F_ic_) arrived at the value of 0.70. A high F_ic _value (close to 1) indicates that the informants use relatively few taxa to manage specific disease conditions as well as consistency in the use of plant species, while a low value indicates that the informants disagree on the taxa to be used in the treatment within a category of illness[11,12].

## Discussion

The fact that 73% of the respondents were aged above 50 years implies that the legacy of the use of traditional medicines to manage HIV/AIDS related infections is in danger of being irrevocably lost if quick efforts are not exerted to document this invaluable knowledge. It is important to note that the sum of plant species used to treat each of the disease conditions as shown in Fig. 2 surpasses the total 75 plant species recorded during the study. This is because many of the plant species reported are used to treat more than one diseases condition.

It is worthy noting that the highest number of the reported herbal remedies was associated with treatment of TB but the highest consensus number of independent reports on the plant remedies against the disease conditions was observed for Herpes zoster. Thus, the degree of informant conformity on a particular plant species in treating a particular disease condition is more important in reflecting the bioactivity potential of the plants than the numerical status of the plants used to treat the condition.

On one hand, the relatively high F_ic _value (0.70) derived suggests that there was a great agreement amongst the respondents on the use of different plant species to manage the reported disease conditions. On the other hand, it reflects the likelihood of presence of bioactive molecules to curtail the various HIV/AIDS opportunistic infections reported. A similar observation was made by Schlage *et al*. [11] who used F_ic _to evaluate the ethnobotanical importance of the medicinal plants of Washambaa in Tanzania. F_ic _is also a crucial tool in establishing a comparative estimation of the level of informant consensus on the use of herbal remedies between culturally different communities [12].

The supremacy of the families Anacardiaceae, Asteraceae, Capparaceae, Clusiaceae, Euphorbiaceae, Lamiaceae, Mimosaceae, Myrtaceae, Papillionaceae and Rubiaceae in the management of the reported conditions could be associated with the presence of certain bioactive secondary metabolites. For example, the families Myrtaceae and Lamiaceae are rich in terpenoids which are biologically responsible for the general improvement and maintenance of body health [17], with a prospective role of boosting the body immunity and consequential potential of managing the reported opportunistic infections. The family Anacardiaceae is rich in tannins, flavonoids and triterpenes which are responsible for prevention of diarrhea, dermal ulcers, general skin eruptions and abdominal pains [18-22]. This may support the pertinent traditional uses of *Ozoroa insignis, Rhus natalensis, R. vulgaris *and *Lannea schimperi *in their respective treatment of skin rashes, Herpes simplex, H. zoster and chronic diarrhea as reported in Table 1.

The ethnomedical uses of some plants described here are consistent with data reported previously. For instance, the traditional use of *Harungana madagascariensis *among the *Igbos *of southeastern Nigeria for the treatment of diarrhea has been reported by Okoli *et al*. [21]. The use of *Psorospermum febrifugum *by the Kamba of Kenya and the Washambaa of Tanzania in the treatment of skin infections [19,23] also concurs with the findings of the present study. Similarly, the use of *Garcinia huillensis *and *Securidaca longipedunculata *to treat Cryptococcal meningitis is consistent with data reported by Mathias [24]. Such a similarity in the cross-cultural usage of plant remedies is a strong indication of the bioactivity potential of the reported plants.

## Conclusion

The information provided forms a strong basis for conservation of the reported remedies, considering that a greater percentage of the plant parts used were roots and stem/bark. Continuous unsustainable harvesting of these plant parts may eventually lead to disappearance of these invaluable resources if early conservation measures are not taken. In future, there would be a need to engage in value adding and standardization of the herbal preparations by developing the necessary dosages and packaging of the herbal formulations. The ethnopharmacological information reported forms a crucial lead for further research to identify and isolate bioactive constituents that can be developed to drugs for the management of the HIV/AIDS opportunistic infections.

## Competing interests

The author(s) declare that they have no competing interests.

## Authors' contributions

DPK was involved in the conception, acquisition and analysis of data, drafting and final revision of the manuscript. HVML designed the research layout, was involved in data analysis and interpretation, and critically revised the manuscript for important intellectual content. KMH and CCJ were involved in the conception, designing of the research and critical revision of the manuscript for important intellectual content. All authors read and approved the final manuscript.
